# ISX9 loaded thermoresponsive nanoparticles for hair follicle regrowth

**DOI:** 10.1016/j.mtbio.2023.100849

**Published:** 2023-11-03

**Authors:** Sapna Sayed, Mehdihasan Shekh, Jiaxing Song, Qi Sun, Han Dai, Vivian Weiwen Xue, Shanshan Liu, Bing Du, Guangqian Zhou, Florian J. Stadler, Guangming Zhu, Desheng Lu

**Affiliations:** a Guangdong Provincial Key Laboratory of Regional Immunity and Diseases, International Cancer Center, Department of Pharmacology, Shenzhen University Medical School, Shenzhen, China; b New Energy Materials Laboratory, College of Materials Science and Engineering, Shenzhen University, Shenzhen, China; c Medical Scientific Research Center, Life Sciences Institute, Guangxi Medical University, Nanning, China

**Keywords:** ISX9, Wnt/β-catenin pathway, Transdermal drug delivery, Nanoparticles, Androgenetic alopecia

## Abstract

There is a high demand for an optimal drug delivery system to treat androgenetic alopecia. Topical application of ISX9, which is a neurogenesis inducer, has been found to stimulate hair follicle (HF) regrowth by upregulating the Wnt/β-catenin signaling pathway, an essential pathway involved in initiating HF growth and development. In the present study, a temperature-sensitive, biopolymer-based, biocompatible, and eco-friendly drug-delivery system was synthesized. This system comprised chitosan-grafted poly(glycidyl methacrylate-*co*-N-isopropyl acrylamide) (Poly(GMA-*co*-NIPAAm)@CS-PGNCS) as the shell component and PF127 as the core polymer. The hydrophobic nature of the PF127 block copolymer efficiently dissolved the partially water-soluble drug, ISX9, and the thermos-responsive shell polymer effectively released the drug at a definite skin temperature. The optimized spherical nanoparticles demonstrated the lowest critical solution temperature (LCST) at 32 ± 2 °C with a diameter of 100–250 nm, which delivered encapsulated ISX9 with greater precision than topical ISX9. In a series of *in vivo* experiments, we demonstrated that ISX9-coated TBNPs upregulated the expression of β-catenin, active β-catenin, Wnt target genes, stemness marker genes, proliferating cell nuclear antigen, HF stem cell markers, and HF markers including VEGF, TGF, and IGF-1 more effectively than topical ISX9. These results suggest that TBNPs could be employed as a platform for effective transdermal delivery of various hydrophobic drugs.

## Graphical abstract

Graphical abstract: Schematic representation of thermos-responsive bi-polymeric nanoparticles (TBNPs) synthesis; (a). Preparation of TBNPs, (b). Chemical structures of raw materials, and (c). A schematic representation of action of ISX9@TBNPs for hair regeneration via the Wnt/β-catenin pathway. ISX9 beleaguered Axin1 and strengthen the LRP6-Axin1 interaction, in this manner causing the stabilization of β-catenin and raised up expression of Wnt, hair and stemmness marker genes for hair regeneration.

**Figure:**
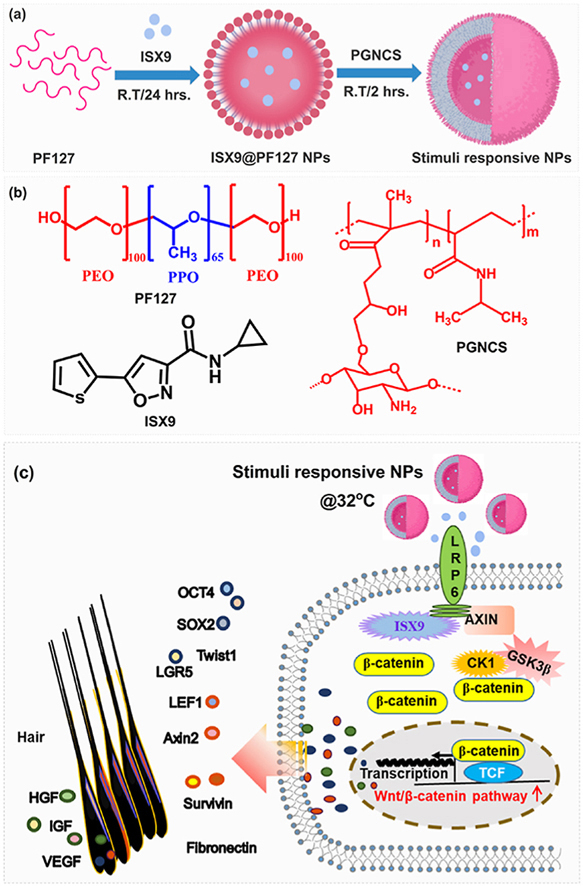

## 1 Introduction

Androgenetic alopecia (AGA) is a persistent hair loss problem, and recent reports indicate a significant increase in the number of people affected by this condition [1]. AGA affects both men and women, but it is more severe in males over the age of 80 and can affect up to 50 % of men in their middle years [2]. Minoxidil (MND), finasteride and Cyproterone acetate are FDA-approved topical treatment for AGA, but it has shown considerable side effects such as inflammation, itching, redness, anxiety, distress, hypertrichosis, weight gain, depression, breast symptoms and sexual dysfunction [3,4]. Isoxazole-9 (ISX9) is a potential alternative to MND [5]. It is a prominent isoxazole derivative known for its role in neuronal growth, maturation, disparity, and proliferation by modulating multiple signaling pathways [[6], [7], [8]]. A recent discovery has indicated that ISX9 may trigger regeneration of hair follicles via stimulation of Wnt/β-catenin signaling. It has been reported that β-catenin is most highly expressed during the anagen phase, which is the period when HF is formed and grows [9]. Although the precise mechanism of ISX9 action is unclear, our recent research indicates that ISX9 activates Wnt/β-catenin signaling and targets Axin1 via its N-terminal region. ISX9 abolishes the association of Axin1 with β-catenin and potentiates the LRP6-Axin1 interaction, leading to the stabilization of β-catenin and upregulation of Wnt target and stemness marker genes. ISX9 shows great promise as a therapeutic agent for alopecia treatment [5].

Accurate dosing of ISX9 is essential to avoid adverse effects, like other medications [10]. Since ISX9 has limited water solubility, only a topical formulation dissolved in organic solvent can promote hair growth [5]. However, long-term usage of organic solvent-based treatments has been shown to cause permanent health issues, such as allergic dermatitis, soreness, and burning sensations of the scalp [11,12]. Additionally, the proportion of transdermal penetration fluctuates considerably with respect to the gender, skin category, establishing the defined and effectual delivery of topical medications challenging [11,13].

To overcome limitations of topical drug delivery, dissolvable and stimuli-responsive nanoparticles are an effective option. Polysaccharide-based biopolymers, such as Chitosan (CS), are ideal for developing drug delivery systems due to their biodegradability, biocompatibility, and mechanical strength for skin penetration [14,15]. CS can also maintain the activity of encapsulated compounds and be modified to exhibit stimuli-responsive behavior such as pH, temperature, and light [14,16,17]. Among the stimuli, thermo-responsive modified CS materials have been extensively studied in biomedical applications [17,18]. Researchers have modified CS with acrylates and polymerized with NIPAAm or directly blended with PNIPAAm [14,19,20]. To increase the effectiveness of biomedical applicability, chemically bonded PNIPAAm chains are preferred. Therefore, our hypothesis is that modified chitosan through GMA copolymerization with NIPAAm, encapsulating the ISX9@PF127 core, can overcome ISX9 delivery obstructions and increase efficiency of delivery across the skin barrier.

Our present study resulted in the development of biocompatible nanoparticles that possess enhanced solubility, stability, and thermos-sensitivity, making them ideal components for efficient drug delivery systems. These nanoparticles were produced using a simple nanoprecipitation method, with PF127 trapping hydrophobic ISX9 and functionalized chitosan coating the drug-loaded PF127 micelles. This ISX9 delivery system was formulated without any exclusive chemical crosslinking. We also assessed the biocompatibility, cytotoxicity, bioavailability, drug release, and *in vitro* cytotoxicity of the ISX9@TBNPs, as well as their effects on hair development in C57BL/6 mice.

## 2 Materials & methods

### 2.1 Materials

The low molecular weight chitosan (degree of deacetylation of 90–95 %, MW: <100 KDa), glycidyl methacrylate (GMA), hydroquinone (HQ), acetic acid (AR, 99.5 %), acetonitrile (AR, 99 %), tetrahydrofuran (THF, AR, 99 %), methanol (AR, 99.8 %) and dimethyl sulfoxide (DMSO, AR, 99 %) were supplied by Macklin Chem. Ltd. (Shanghai, China). Pluronic F-127 (PF127, Bio reagent grade, Density: 1.06 g/cm^3^) was purchased from Sigma-Aldrich Co. (USA). Acetone (AR, 99 %) was purchased from Kunshan Jinchang Reagent (SJR) Co. Ltd. (Kunshan, China). ISX9 was supplied by TargetMol Chemicals Inc. (Wellesley Hills, Massachusetts). HyClone™ Water and HyClone™ Phosphate Buffer Saline (PBS) were purchased from GE Healthcare Life Sciences (Marlborough, MA, USA). Acetonitrile (HPLC grade) was obtained from Honeywell (Charlotte, NC, USA). The human embryonic kidney 293T (HEK293T), Human keratinocytes (HaCaT) and the mouse embryonic fibroblasts (NIH3T3) were purchased from ATCC (Manassas, VA, USA). Dulbecco's modified Eagle's medium (DMEM), fetal bovine serum (FBS), 1 % penicillin-streptomycin (GIBCO), antibiotic-antimycotic (AA), and trypsin were purchased from Thermofisher (Waltham, MA, USA). Cell Counting Kit-8 (CCK-8) was obtained from Dojindo Laboratories (Kumamoto, Japan).

### 2.2 Methods

#### 2.2.1 Preparation of temperature sensitive biodegradable nanoparticles

The synthesis of temperature-sensitive biopolymeric nanoparticles involved two steps. Firstly, chitosan (CS) was modified with GMA and polymerized with NIPAAm to create a shell component called PGNCS. Secondly, the shell was coated onto a PF127 core. The following subsections provide a brief description of the procedure.

#### 2.2.2 Modification of CS with GMA (GCS)

The process of modifying the CS using GMA was carried out following the report, but with minor adjustments. First, low molecular weight CS (1 g) was stirred in 0.4 M acetic acid (AcOH) at room temperature. Then, GMA (2.4 mmol) and hydroquinone (0.001 mM) solution in 0.05 M potassium hydroxide (10 mL) were added, and the nitrogen gas was purged in the reaction mixture. The reaction mixture was stirred and heated in a preheated oil bath at 65 °C for 2 h under constant stirring and continuous nitrogen flow. The reaction mixture may turn white, indicating polymerization of GMA and precipitation of CS at neutral pH. To further purify, the reaction mixture was slowly poured into acetonitrile to remove the polymerized GMA. The product was dried under vacuum and then dissolved in acidic water (pH 5.0). The GCS solution was dialyzed (MWCO: 12 k-14 k Dalton) for at least 1.5 days to remove lower molecular weight chains of GCS. The light-yellow transparent solution was freeze-dried at −60 °C using a freeze-drying machine. The fibrous product of GCS was collected and stored in a refrigerator at 15 °C. The degree of grafting was confirmed through gravimetric analysis.

#### 2.2.3 Copolymerization of GMA@CS with NIPAAm (PGNCS)

To achieve temperature sensitivity in GMA@CS and ensure that the temperature sensitivity of the shell polymer is similar to skin temperature (32.5 °C), different molar amounts (1:0.5, 1:1, 1:1.5, 1:2) of NIPAAm were copolymerized with GCS using the free radical polymerization technique. The molar ratio was determined with respect to the GMA units. GCS was dissolved in 50 mL 0.4 M AcOH and the NIPAAm solution (in water) was slowly added under nitrogen purging. The reaction mixture was heated in a preheated oil bath for 3–4 h at 65 °C after adding 0.05 % wt of ammonium persulfate (NH_4_)_2_S_2_O_8_). The reaction mixture was then poured into THF solvent, filtered using a vacuum filtration technique, and dried under vacuum at room temperature. The copolymer was further purified by dissolving it in water and dialyzing for one day to remove lower molecular weight chains. The resulting poly(GMA-co.-NIPAAm)@chitosan (PGNCS) was solidified through freeze-drying and stored in a refrigerator at 15 °C. This same procedure was followed to prepare other copolymers with different amounts of NIPAAm, denoted as PGNCS-X, where X equals the amount of NIPAAm (i.e., 0, 0.5, 1, 1.5, 2).

#### 2.2.4 Preparation of PF127 core nanoparticles

The procedure for preparing PF127 nanoparticles involved dissolving 20 mg of PF127 in 1 mL of acetone, stirring the solution for 1 h at room temperature, and then adding the PF127@acetone solution dropwise into 4 mL of double distilled water with stirring for 2 h. The resulting mixture was left overnight in a fume hood to remove the acetone, followed by centrifugation at 2500 RPM, collect the supernatant, and left over for 2 h. The PF127 nanoparticles were collected for further use. A similar method was employed for producing drug-loaded PF127 nanoparticles, where different amounts of ISX9 drug (0.25 %, 0.5 %, 1.0 % wt relative to the core polymer) were added to 20 mg of PF127@acetone solution (1 mL) and processed in the same manner. The nanoparticles were stored at 15 °C until further use.

#### 2.2.5 Preparation of thermos-responsive bipolymeric nanoparticles (TBNPs)

In this study, PF127 was employed as the core material, while functionalized chitosan (PGNCS) was used as the shell material in varying amounts. First, a fresh solution of PF127 NPs (20 mg PF127, 4 mL) was stirred at room temperature. Then, different amounts of PGNCS (20 mg, 30 mg, and 50 mg) were added dropwise to the PF127 solution, and the mixture was stirred for 2–3 h. The solution was then centrifuged at 1500 rpm for 10 min to remove larger reactant particles. The resulting solution was filtered multiple times through a 0.22 μm filter paper using vacuum filtration and stored at 15 °C. The different NPs obtained were designated as TBNPs-Y, where Y = 1 to 3 corresponds to the amount of PGNCS used.

#### 2.2.6 In-vitro drug release

The release profile of ISX9 drug from TBNPs-1 having 1 wt % was evaluated in PBS solution (0.1 M, pH 7.0) at temperatures of 32 ± 2 °C and 25 ± 2 °C. A specific amount of ISX9 loaded PGNCS was dispersed in 5 mL of 0.1 M PBS solution and sealed in a dialysis bag (MWCO: 3500 Da). The bag was immersed in 100 mL of fresh 0.1 M PBS solution at a constant temperature of 200 rpm. At different times, various amounts of the test media were added after removing the 5 mL solution. The cumulative amount of released drug was calculated using equation (1):

(1) Cumulative amount of release drug = C_i_/C_0_ X 100 % …

where C_i_ is the amount of the drug that is released at a specific interval of time, and the amount C_0_ is the amount of the loaded drug. The temperature was recorded in degrees Celsius (°C).

The encapsulation efficiency and loading capacity were evaluated using foowing equations (2), (3), respectively.

(2) $$\text{Encapsulation}\;\text{efficiency}\;\left(\%\right)=\frac{The\;total\;amount\;of\;drug-unbounded\;drug}{The\;total\;amount\;of\;drug}\times 100\ldots$$

(3) $$\text{Loading}\;\text{capacity}\;\left(\%\right)=\frac{The\;total\;amount\;of\;drug-unbounded\;drug}{weight\;of\;the\;nanoparticles}\times 100\ldots$$

### 2.4 Characterization

The chemical structure of the modified CS, PGNCS copolymers was analyzed using FTIR (FT-IR, Shanghai, China). All samples were scanned from 4000 to 450 cm^−1^ wavenumber at a resolution of 0.4 cm^−1^ with 64 scans. The ^1^H NMR spectra were recorded using trimethyl silane as the reference (nuclear magnetic resonance spectrophotometer, Switzerland). LCST was determined using the differential scanning calorimetry (DSC) method (Q2000 differential scanning calorimeter, TA instruments, Delaware, USA). The test samples were dispersed in water (2 mg/mL) and heated under a nitrogen gas atmosphere from 20 °C to 60 °C with a heating rate of 3 °C/min in an alumina crucible. The DSC curves were normalized, and TRIOS™ software was used to determine the LCST. Nanoparticle morphologies were confirmed using HR-TEM. TEM samples were prepared by dispersing the nanofibers in ethanol to create a uniform suspension, and then placing a drop of the solution on a carbon-coated copper grid. The grid was dried at a temperature of 40 °C for 24 h. The hydrodynamic diameter and zeta potential of the nanoparticles were determined using a Malvern Zeta sizer Nano instrument at 25 °C in water at a concentration of 0.1 mg/mL. The ISX9 drug release profile of TBNPs was evaluated in 0.1 M PBS solution (pH 7.0), and the released ISX9 from nanoparticles was detected on an ultraviolet–visible spectrophotometer (UV 2450, Shimadzu, Tokyo, Japan).

### 2.5 In vitro cell activity, viability and cytotoxicity analysis of ISX9@TBNPs

For activity assessment of ISX9 encapsulation in TBNPs, HEK293T cells were well-developed in 10-cm dishes. After 24 h, attaining 50 % confluence, cells were transfected with 5 μg of SuperTOPFlash or SuperFOPFlash reporters, carrier DNA pcDNA3 and control-plasmid pCMX-β-gal (1 μg of β-galactosidase, β-gal). Next, later 24 h, cells were dispersed in 96-well microtiter plates. Finally, transfected cells were treated with 5 μM of ISX9, topical ISX9, and ISX9@TBNPs, DMSO, topical control (50 % [v/v] ethanol, 30 % water and 20 % propylene glycol), and TBNPs, for their effect. By means of a microtiter plate luminometer, later on cells were lysed and events were noticed via luciferase by means of a luciferase assay kit (Promega Cat# E1501, Madison, WI, USA). Using a β-galactosidase as an inner control, the tenets from luciferase assay were normalized for dissimilarities in transfection efficiency.

To assess the biocompatibility of ISX9, HaCat and NIH3T3 cells were analyzed for apoptosis using flow cytometry. The cells were first washed twice with PBS and then once with Annexin V buffer (BD Biosciences), stained with Annexin V-FITC (BD Biosciences) and PI (BD Biosciences) in the dark at 4 °C for 15 and 20 min, respectively. The samples were then analyzed using a FACSAria II system (BD Biosciences) and the results were analyzed using FACSDiva software.

For cytotoxicity analysis of TBNPs, HaCaT and NIH3T3 cells were cultured in DMEM supplemented with 10 % FBS and plated at a density of 10,000 cells per well in a 96-well plate, followed by incubation at 37 °C overnight. Various concentrations of nanoparticles including PluNPs, GMA@CS, TBNPs-1, TBNPS-2 and TBNPS-3 were then added to the cells and incubated for 24 h. Cytotoxicity was assessed using the CCK-8 assay at a wavelength of 450 nm on a microplate reader (TECAN, Männedorf, Switzerland) with triplicate analyses conducted for accuracy.

### 2.6 In vivo hair growth model

A hair growth model was prepared *in vivo* using female C57BL/6 mice (6 weeks old, purchased from Nanjing Biomedical Research Institute). All animal care guidelines were followed according to the rules and regulations of Shenzhen University's Animal Research Ethics Committee. Anesthesia was administered only during hair depilation, treatment, observation, and photography of fresh hair regrowth. The hair regrowth experiments were conducted with permission from the Animal Research Ethics Committee. Mice were acclimatized to their environment for one week, with a room temperature of 20 ± 2 °C, humidity level of 50 ± 5 %, 12 h light and 12 h dark periods, and free access to normal feed and tap water.

After one week of acclimating to the mice's diet and using a complete randomization method, we created four groups labeled as 1, 2, 3, 4 and 5 (1: Topical control (50 %[v/v] ethanol, 30 % water and 20 % propylene glycol, 2: Topical ISX9 (1 % ISX9), 3:ISX9@PluNPs, 4: TBNPs, 5: ISX9@TBNPs) with 5 mice in each group. After inspection the telogen phase of the mice skin, samples (300 μL of solution containing 45 mg/kg ISX9) were applied topically on the clean-shaven dorsal skin for three days per week for 24 days. The dorsal skin parts of the mice were frequently monitored and documented with a digital camera every week.

### 2.7 Hair cycle analyses and hair weight analysis

The analysis of the hair cycle focused on skin pigmentation levels and hair shaft growth, as previously reported. Clean-shaved hair-coat-recovery values were measured based on skin pigmentation levels ranging from 0 to 100 % [21]. For instance, 0 % indicated no hair growth (and no pigmentation); 50 % represented full-length hair growth covering half of the dorsal skin (or pigmentation level on 100 % of the dorsal skin area without any hair shafts); 70 % indicated full-length hair growth on approximately 70 % of the dorsal skin (or pigmentation level covering 100 % of the dorsal skin along with 30–40 % hair shafts); 100 % indicated fresh full-length hair growth.

After 24 days, all mice were sacrificed with respect to their groups via cervical dislocation. The next step in assessing hair regrowth is to determine the weight of hair removed from the 1 cm^2^ surface using analytical weights. Placed the collected hair on an aluminum sheet and mark it to determine whether the hair belongs to the test area or the control area. The weight of hair is expressed in mg/cm^2^ [22].

### 2.8 Histology and immunohistochemistry analyses

The excised skins were fixed in 10 % neutral buffered formalin and embedded in paraffin using an established protocol for histological analysis. The paraffin blocks were sectioned at 5 μm thickness using a microtome from Leica, Wetzlar, Germany, stained with hematoxylin and eosin (H&E), and examined using a microscope (DMI 3000B, Leica, Wetzlar, Germany) to evaluate new hair follicle formation. For immunohistochemistry, primary antibodies such as anti-Ki-67 (1:200, Cell Signaling Technology Cat# 12202, RRID: AB_2620142), anti-active β-catenin antibody (1:100 Cell Signaling Technology Cat#8814, RRID: AB_11127203), and anti-β-catenin (1:100 Cell Signaling Technology Cat# 9562, RRID: AB_331149) were used.

### 2.9 Western blot analysis

The smooth-shaved dorsal tissue samples from each group were minced using RIPA buffer (0.1 % SDS, 150 mM NaCl, pH 7.2, 10 mM Tris, 1 % sodium deoxycholate, 5 mM EDTA, 1.0 % Triton X-100). Equal amounts of protein were separated on SDS polyacrylamide gels and transferred onto PVDF membranes (Millipore, USA). The membranes were blocked with 5 % non-fat milk in PBS for 1 h, and then incubated overnight at 4 °C with antibodies against active β-catenin, β-catenin, K17, Axin2, LEF1, survivin, LGR5, SOX2, pLRP6, LRP6, GSK3β, GAPDH, pGSK3β and Ki67. After washing, the membranes were incubated with HRP-conjugated goat anti-rabbit or anti-mouse IgG at room temperature for 1 h. The immunoblots were visualized using the ECL Plus-Western-Blotting substrate (Thermo Fisher Scientific, Cat# 32132) and Chemiluminescent Imaging System (Tanon 5200, Shanghai, China).

### 2.10 RNA extraction and real-time PCR analyses

The RNA was extracted using RNAiso Plus according to the supplier's guidelines. The extracted RNA was then converted to cDNA using the Primescript RT Kit following the kit's instructions. The cDNA was used for quantitative PCR analysis on an ABI Prism 7300 Real-Time PCR System with qPCR Master Mix. The primer sequences are listed in Table S1.

### 2.11 In vivo toxicity evaluation

At the conclusion of the *in vivo* animal model experiments, the cytotoxicity of TBNPs and ISX9@TBNPs was evaluated by examining major organs, including the heart, lungs, spleen, liver, and kidneys, for potential toxicity using histology.

### 2.12 Statistical analysis

All measurements were taken in triplicate and reported as mean ± standard deviation (SD). A statistical analysis comparing two groups was conducted using Student's t-test with significance set at p < 0.05.

## 3 Results and discussion

### 3.1 Thermo-responsive bi-polymeric nanoparticles synthesis

**Fig. 1:**
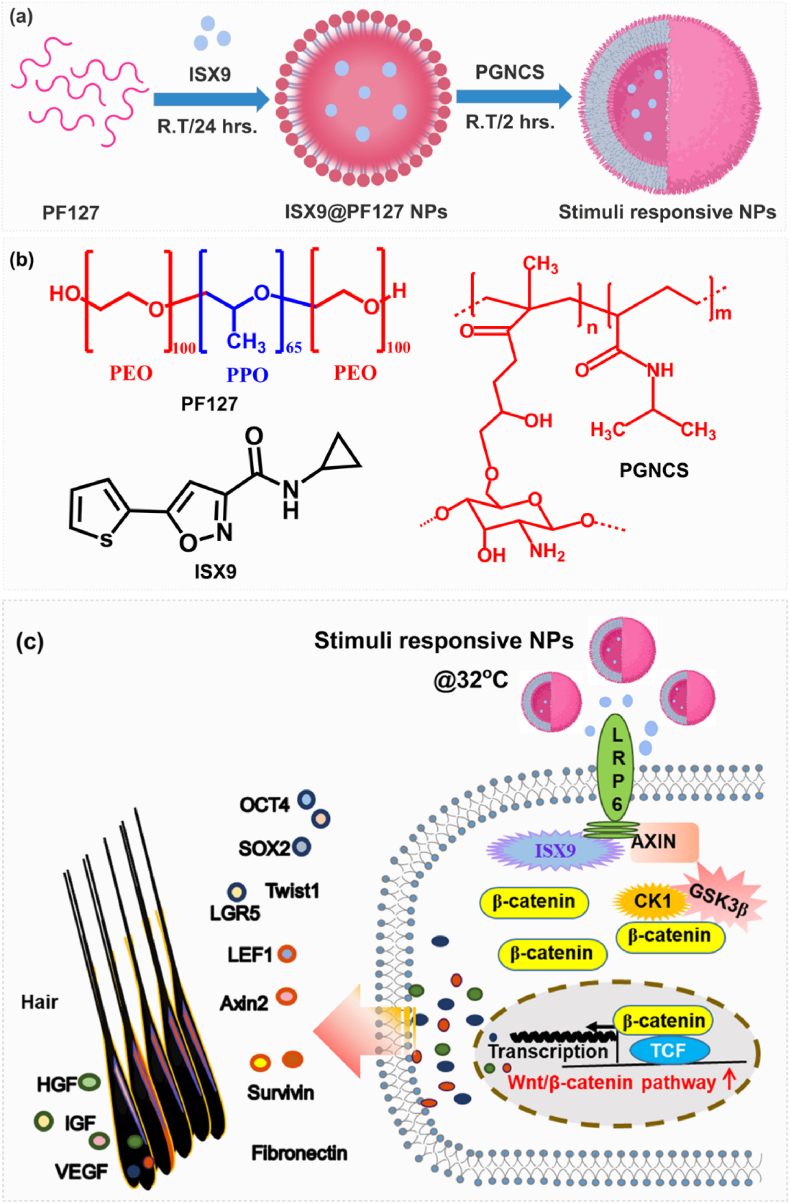
Schematic representation of thermos-responsive bi-polymeric nanoparticles (TBNPs) synthesis; (a). Preparation of TBNPs, (b). Chemical structures of raw materials, and (c). A schematic representation of action of ISX9@TBNPs for hair regeneration via the Wnt/β-catenin pathway. ISX9 beleaguered Axin1 and strengthen the LRP6-Axin1 interaction, in this manner causing the stabilization of β-catenin and raised up expression of Wnt, hair and stemmness marker genes for hair regeneration.

The present study outlines a straightforward approach to synthesize biopolymeric nanoparticles from two distinct polymer matrixes with different properties, such as self-assembly and thermo-responsiveness [19]. Fig. 1 illustrates the formation of TBNPs, where the hydrophobic drug was dissolved in PF127 core, while the thermoresponsive shell, i.e., poly(GMA-co.NIPAAm)@CS (PGNCS), covered the ISX9@PF127 core (Fig. 1). The use of PF127 was justified due to the block copolymer's ability to deliver hydrophobic (water-insoluble) drugs by forming micelle-like structures around the drug molecules [23]. The coating of a thermoresponsive polymer (chitosan, GMA, and NIPAAm) with a positively charged surface is ideal for effective drug delivery systems for the skin. The thermos-responsive shell was fabricated by modifying chitosan (CS) through glycidyl methacrylate (GMA) followed by copolymerization with N,N-dimethyl-isopropyl acrylamide (NIPAAm) (Fig. S1). The obtained shell polymer contained different ratios of GMA@CS (GCS having a 13–14 % of GMA units on CS polymer backbone) and NIPAAm (i.e., 1:0.5, 1:1, 1:1.5, 1:2). The structure of GCS and poly(NIPAAm-*co*-GMA@CS) (PGNCS) was confirmed by FT-IR and ^1^H NMR spectroscopic techniques. Fig. S2 shows the ^1^H -NMR spectra of CS, GCS, and PGNCS. The identical repeating units of CS polymer showed the multiplates of -O**H** (3.0–3.6 *δ* ppm), -N**H**- (7.9 *δ* ppm), -N**H**_**2**_ (4.7 *δ* ppm), -N-C**H**- (3.9–4.3 *δ* ppm), -*O*-C**H**- (4.8–5.1 *δ* ppm), -C**H**_**2**_-O (2.4 *δ* ppm) and –CO–C**H**_**3**_ (1.8 *δ* ppm) protons in ^1^H NMR spectrum. After grafting of GMA on CS chain, new protons signals were found regarding to the GMA chain. The multiplates corresponded to the -*O*–CH_2_–, -C-O**H**-, -*O*-C**H**-, -C-C**H**_**2**_-CO-, –C=C–C**H**_**3**_-, and -C**H**=C**H**- were shown peaks at 3.8–3.9 *δ* ppm, 3.5 *δ* ppm, 3.7–3.8 *δ* ppm, 2.9 *δ* ppm, 1.5–1.6 *δ* ppm and 5.3 *δ* ppm-5.8 *δ* ppm, respectively. The presence of new signals related to alkene (-CH=CH-) and significant changes between 3.0 and 3.6 *δ* ppm confirmed the successful grafting of GMA on hydroxyl of CS. The grafting reaction occurred on the –CH_2_-OH group of CS in acidic media, rather than the -NH_2_ (amine) group. Further, the absence of peaks at strong field (0.5–1.2 *δ* ppm) confirmed that no polymerization occurred during the grafting of GMA on CS. Additionally, the ^1^H NMR spectrum of PGNCS-1 showed similar signals to GCS, with minor shifting. The significant changes in ^1^H NMR were observed as the multiplates between 0.5 and 1.5 *δ* ppm.

The structure of the different poly(NIPAAm-co.-GMA@CS) polymers was confirmed using FT-IR (Fig. S3a). The spectra showed characteristic bands related to the amine, hydroxyl, and alkyl stretching vibrations between 3500 and 3200 cm^−1^ and 2950-2850 cm^−1^, respectively. Major changes were seen in the vibration bands of C=O (1680 cm^−1^), -N-H (1450 cm^−1^), C–N (1370 cm^−1^), and C–O (1200-1050 cm^−1^). With an increasing concentration of NIPAAm, the intensity of these bands also increased, which further confirmed the successful copolymerization. The polymerization of GCS and NIPAAm was carried out in aqueous media, and as the NIPAAm concentration increased, the final reaction mixture showed lower whiteness to intense whiteness (above R.T.). This confirmed that the lowest critical solution temperature (LCST) of PGNCS was tuned with changing the ratio of NIPAAm. The DSC curves with LCST values for PGNCS were shown in Fig. S3b, and PGNCS-1 with a ratio of 1:1 showed 32 ± 2 °C of LCST, which was well-suited for topical drug delivery and efficient penetration in the skin. Therefore, PGNCS-1 was used for further coating of PF127 nanoparticles.

**Fig. 2:**
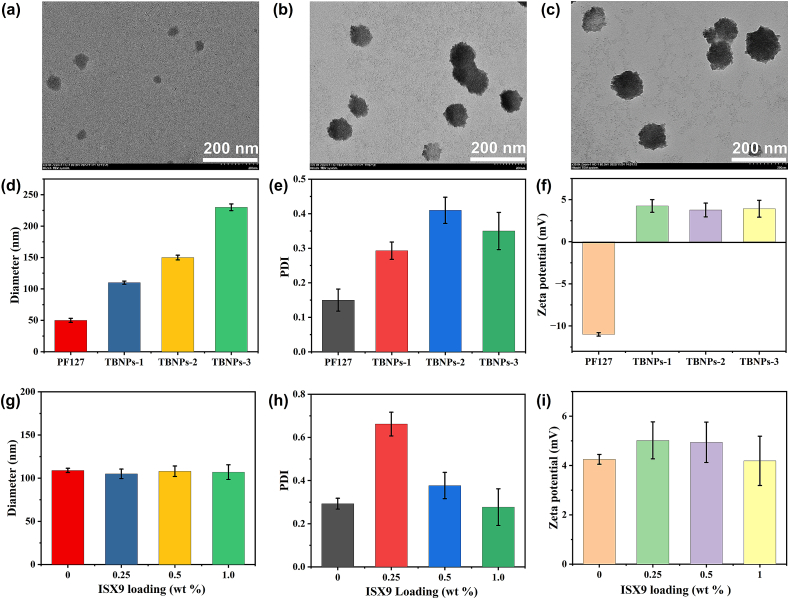
TEM images of nanoparticles- (a) PF127, (b). TBNPs-1, (c) TBNPs-2; Physicochemical properties of TBNPs having different amount of PGNCs-1 polymer as shell material with respect to the PF127 amount (i.e., 20 mg–50 mg), (d) hydrodynamic diameters, (e) polydispersity index (PDI), (f) zeta potential; and physicochemical properties of TBNPS-1 having different amount of ISX9 (i.e., 0.25 % wt to 1 % wt), (g) hydrodynamic diameters, (h) polydispersity index (PDI), and (i) zeta potential.

To create the core structure, PF127 was dissolved in acetone and added to distilled water. This led to the self-assembly of polymer chains into micelle-like structures with a diameter of 40–60 nm (Fig. 2a & d), with a negative surface charge of −10 mV (Fig. 2f & Fig. S4) [[24], [25], [26]]. The resulting PF127 NPs were then coated with varying amounts of PGNCS-1, resulting in the formation of core-shell nanoparticles (i.e., TBNPs-1, TBNPs-2, & TBNPs-3) with hydrodynamic diameters of 100 nm ± 4.5, 148 nm ± 5.8 and 230 nm ± 6.4, respectively while their PDI varied between 0.3 and 0.4 with positive surface charge (Fig. 2d–f & S4). It was clearly seen from Fig. 2d and S4, the hydrodynamic diameter of TBNPs were increased with increasing of coating polymer ratio while the PDI also elevated which may be due to the presence of other small artifacts or water bubbles which generally found in precipitation method and nanospheres systems [27]. Interestingly, surface charge of PGNCS-X (i.e., (X = 0.5, 1, 1.5, 2) and TBNPs nanoparticles were demonstrated the negligible diffrences which suggesting that amine group of chitosan on PGNCS polymer were responsible for positive surface charge (Fig. 2f & Fig. S5). The dense inner morphologies of PGNCSwere visible in TEM microscopy (Fig. 2a and b).

ISX9, a Wnt-specific activator, was used as a hair growth stimulant. Since it is insoluble in water, it was dissolved in acetone with PF127 and then added to distilled water. The resulting solution was stirred, centrifuged, and filtered through a vacuum filtration machine to obtain drug-loaded nanoparticles. The different drug-loaded TBNPs-1 NPs had similar hydrodynamic diameters, PDI, and zeta potential (Fig. 2g–i). We have used TBNPS-1 and 1 wt % ISX9@TBNPS-1 nanoparticles for *in-vitro* and *in-vivo* experiments.

### 3.2 Activity and biocompatibility of TBNPs

**Fig. 3:**
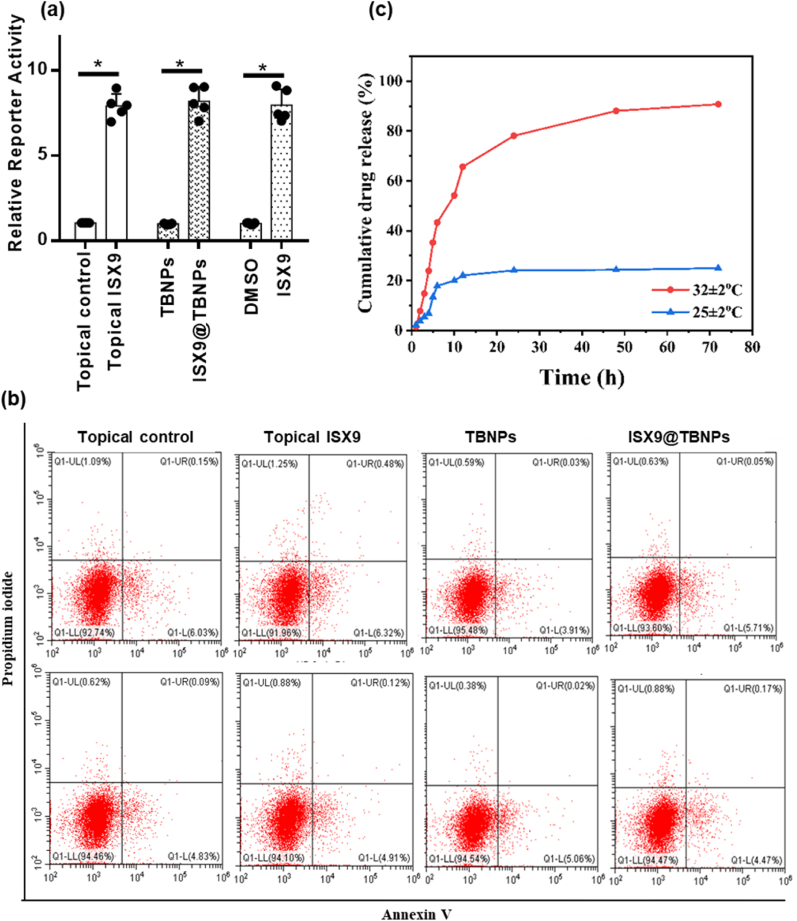
**ISX9 retains its activity and biocompatibility in TBNPs-encapsulated form.** (a) HEK293T cells carrying a Wnt responsive luciferase reporter (SuperTOPFlash) were treated with topical control, topical ISX9, TBNPs, ISX9@TBNPs, DMSO and ISX9. The cells were lysed and luciferase activity was quantified. Values are means ± SD. *P < 0.05, significantly different from the topical control and TBNPs. (b) Apoptosis analysis of HaCat and NIH3T3 cells after topical control, topical ISX9, ISX9@TBNPs and TBNPs treatments via Flow cytometry analysis (FACS). (c) Cumulative release profiles of ISX9 from ISX9@TBNPs nanoparticles at 25 ± 2 °C and 32 ± 2 °C (skin temperature). Data were expressed as mean ± SD, n = 6.

The cytotoxicity of empty nanoparticles was evaluated *in vitro* on HaCaT cells and NIH3T3, with PF127, GMA@CS, TBNPs-1, TBNPs-2, and TBNPs-3 injected at various concentrations. No obvious cytotoxicity was observed, with over ≥96 % cell viability, as shown in Figs. S7(a and b). The activity of ISX9@TBNPs nanoparticles was tested using a cell-based SuperTOPFlash reporter system to measure their ability to activate the Wnt/β-catenin pathway. ISX9, topical ISX9, and ISX9@TBNPs increased reporter activity compared to DMSO, topical control, and TBNPs, suggesting no significant loss of activity during encapsulation, as depicted in Fig. 3a. The biocompatibility of encapsulated ISX9 nanoparticles was assessed by staining HaCat cells with annexin V and propidium iodide (PI) and conducting FACS. The treatments of topical control, topical ISX9, TBNPs, and ISX9@TBNPs maintained 92.7 %, 91.6 %, 95.4 %, and 93.6 % of viable HaCat cells and 94.46 %, 94.10 %, 94.54 %, and 94.47 % of viable NIH3T3 cells, respectively. FACS results indicate that ISX9 did not induce apoptosis in HaCat and NIH3T3 cells Fig. 3b.

### 3.3 In vitro drug release

The drug loading capacity of TBNPs-1 (having 1 wt % drug) and encapsulation efficiency were 82 % and 80 %, respectively. To ensure that the temperature sensitivity of the shell polymer matched the skin temperature (32.5 °C), we used copolymerization of GCS nanoparticles system with NIPAAm. We employed free radical polymerization to copolymerize NIPAAm and GMA units. We investigated the release profiles of ISX9 from TBNPs at both 32 ± 2 °C and 25 ± 2 °C as shown in Fig. 3c to evaluate its release efficiency. ISX9 was rapidly released in the first 10–15 h followed by sustained release in the subsequent hours at both temperatures. The release of ISX9 was faster at 32 ± 2 °C, owing to the temperature-sensitive property of TBNPs, which resulted in rapid release at skin temperature. The cumulative release amount of ISX9 for 12 h was 63 % at 32 ± 2 °C and <20 % at 25 ± 2 °C. PF127@TBNPs had 82 % drug loading capacity and enabled controlled and sustained drug release of ISX9. To support the role of PNIPAAm and PF127 in drug delivery system, we have also carried out some controlled release experiments where we have chosen four different systems; (1). PF127 as core while poly(GMA)@CS as shell polymer, (ii). Polyethylene glycol as core polymer while PGNCS as shell polymer; (iii). bare PF127; and (iv). Bare PGNCS. The obtained results are shown in Fig. S6. Results showed that PF127@PGCS, PEG@PGNCS, and PGNCS have showed sustained drug release profile within 72 h while PF127 showed burst release within 4–5 h at both temperatures. Similarly, TBNPs have showed 90 % of drug release within 24 h at 32 ± 2 °C temperature while approximately 50 % (at 24 h) of drug released at room temperature which suggested that presence of chitosan microstructure still controlling the drug release at ambient temperature as well. The PF127@PGCS showed 40 % of drug release with 24 h while PEG@PGNCS showed 60 % of drug release which possibly related to the presences of thermos-responsive shell in PEG@PGNCS system. Herein, overall, concentration of PF127 in drug system is lower than 1 % which is possibly very lower for thermos-responsive behaviour [25,28]. Moreover, ISX9@PGA/PGNCS, ISX9@PF127/PGCS, PF127 and PGNCS were showed the 88 %, 79 %, 70 % and 83 % of drug loading capacity while 86 %, 79 %, 79 %, and 88 % of encapsulation efficiency. Overall, these findings indicate that TBNPs could be an essential and useful drug delivery system for other hydrophobic drugs [25,28]. Moreover, ISX9@PGA/PGNCS, ISX9@PF127/PGCS, PF127 and PGNCS were showed the 88 %, 79 %, 70 % and 83 % of drug loading capacity while 86 %, 79 %, 79 %, and 88 % of encapsulation efficiency. Overall, these findings indicate that TBNPs could be an essential and useful drug delivery system for other hydrophobic drugs.

### 3.4 *In vivo* efficacy of ISX9@TBNPs on hair growth

**Fig. 4:**
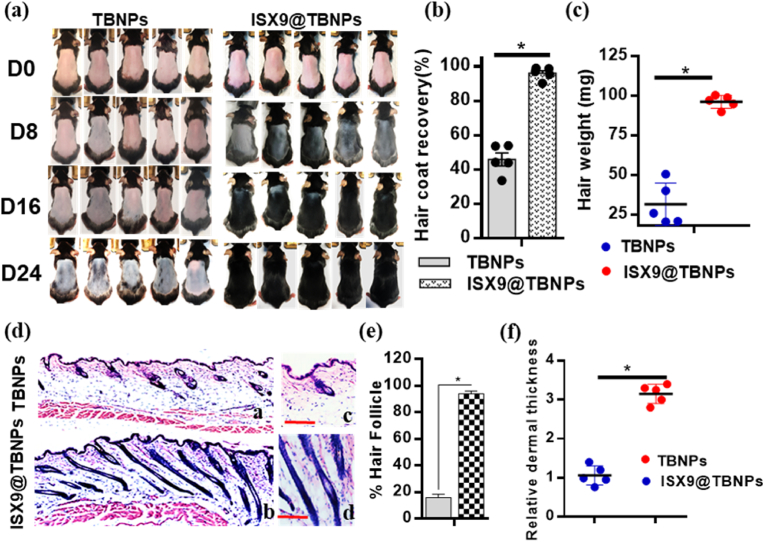
***In vivo* effectiveness of ISX9 loaded nanoparticles in C57BL/6 mice.** C57BL/6J mice in the telogen phase (7 weeks old, female) were depilated. TBNPs or ISX9@TBNPs, were topically applied thrice a week to the dorsal skin for 24 days (n = 5 per group). (a) Representative photos of mice showed hair regrowth on 0, 8, 16, 24th day treated with different combinations: TBNPs or ISX9@TBNPs. (b) Quantitative measurements of hair coat recovery at the designated area at 24th day. (c) Gross analyses of weight of regrown hair in different groups treated at 24th day. Data shown were representative of five independent experiments (*n* = 5). Values are means ± *SD*. *P < 0.05, significantly different from the TBNPs. (d) H&E stained dorsal skin at 24th day in different groups (a, b). Also c, & d were the enlargements of the framed area in a, and b respectively. Scale bar = 100 μm. (e) Quantitative assessment of the relative integer of HF relative to H&E stained dorsal skin (n = 5). (f) Relative dermal thickness among mentioned groups at 24th day. Data are expressed as mean ± *SD* (n = 5). *P < 0.05 significantly different to control.

The results of the study indicate that the ISX9@TBNPs nanoparticles were effective in promoting hair growth in a mice model of depilation. The mice group treated with ISX9@TBNPs showed complete hair regrowth by the 24th day, with 100 % hair regrowth observed. In comparison, the topical ISX9, topical control, ISX9@PluNPs and TBNPs groups did not show such a significant effect. The body weights of all groups were similar, indicating that there were no significant side effects of ISX9 treatment. The changes in hair weight were measured and accounted for the weight of the corresponding hair regrowth in each group after treatment on the 24th day (Fig. 4a–d & S8 a-c). These results suggest that the ISX9@TBNPs nanoparticles could be a potential therapeutic option for hair regeneration and restoration.

For additional assessment of hair growth, the excised dorsal skins in the groups were subjected to H&E assay. As shown in Fig. 4d, S8d-e, both the ISX9@TBNPs and topical ISX9 groups revealed a higher number of hair follicles than the ISX9@PluNPs, TBNPs and topical control groups. Most importantly, the ISX9@TBNPs group had more hair follicles percentage compared to empty nanoparticles TBNPs (Fig. 4e and f). This difference between topical ISX9 and ISX9-TBNPs was credited to the dual-functioning of the drug delivery system, which aimed for high solubility, temperature sensitivity, and efficient release of ISX9 upon the skin.

Based on these findings, we can conclude that polymer-based bipolymeric nanoparticles have considerable potential as a safe and effective platform for transdermal distribution of numerous hydrophobic drugs *in vivo* [29]. After thorough investigation, chitosan-based thermos-responsive nanoparticles were found to be an effective carrier of ISX9 that safely passed the small molecule into the skin layers, causing an excellent hair growth effect, and lacking any conspicuous negative side effects in a mice model. The most significant contribution of our study is the demonstration for the first time that thermos-sensitive NIPAAm-coupled chitosan is the most suitable thermos employing formulation of chitosan nanoparticles in addition to improving topical delivery of hydrophobic drugs [19].

**Fig. 5:**
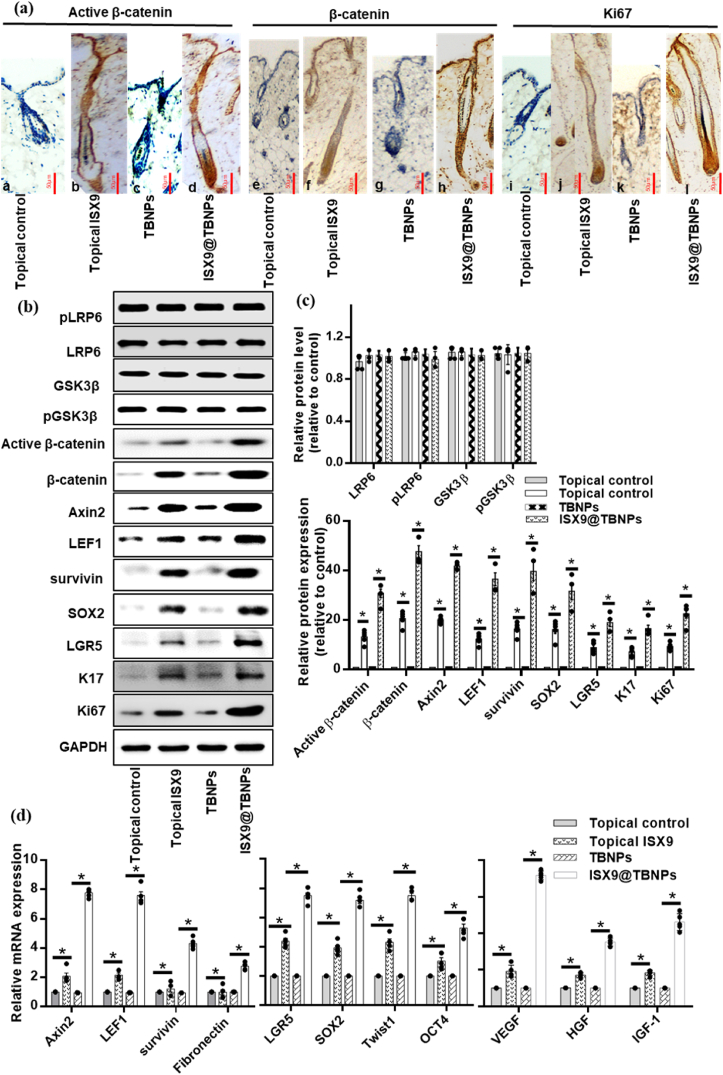
**ISX9@**TBNPs **promotes the expression of hair regrowth-related markers in C57BL/6J mice.**C57BL/6J mice in the telogen phase were depilated. Topical control, topical ISX9, TBNPs and ISX9@TBNPs were topically applied thrice a week to the dorsal skin for 24 days (n = 5 per group). (a) Immunohistochemistry (IHC) staining for active and total β-catenin, and Ki67 using the dorsal skin of mice treated with topical control, topical ISX9, TBNPs and ISX9@TBNPs at 24th day. Scale bars = 50 μm. (b) Immunoblotting analyses for pLRP6, LRP6, GSK3β, pGSK3β, active β-catenin, β-catenin, Axin2, LEF1, survivin, SOX2, LGR5, Keratin 17 and Ki67 on 24th day. (c) Protein bands for pLRP6, LRP6, GSK3β, pGSK3β, active β-catenin, β-catenin, Axin2, LEF1, survivin, SOX2, LGR5, Keratin 17 and Ki67 were quantified by densitometry and graphically presented. (d) The expression of the indicated genes, Axin2, LEF1, survivin, Fibronectin, LGR5, SOX2, Twist1, OCT4, VEGF, HGF and IGF-1 in each treatment group was measured using real-time PCR analysis on 24th day. The relative expression levels of mentioned genes were quantiﬁed after normalization to GAPDH. Data shown were representative of five independent experiments (n = 5). Values are means ± SD. *P < 0.05, significantly different from the topical control and TBNPs; one-way ANOVA followed by Dunnett's *t*-test.

We expanded our analysis by dividing the dorsal skin sections in order to compare the hair regrowth characteristics among different groups. The mice treated with ISX9@TBNPs had the highest number of hair follicles and increased dermal thickness compared to the mice treated with topical ISX9, topical control, ISX9@PluNPs and TBNPs treatments (Fig. 4e and S8e). The histomorphometric analysis of hair follicles also confirmed that ISX9@TBNPs treatment significantly stimulated the telogen-to-anagen shift compared to ISX9 dissolved in organic solvent. Additionally, an immunohistochemistry study supported the effects of ISX9@TBNPs for hair regrowth in tissues from the shaved region. By examining immunohistochemical staining, ISX9@TBNPs was found to be the most effective at regulating dermis and hair follicle morphogenesis at all stages of the process, followed by topical ISX9, as compared to topical control and TBNPs. A significant increase in the expression of active β-catenin and β-catenin was observed in ISX9@TBNPs and topical ISX9 groups compared with TBNPs and topical control. It was also revealed that Ki67, an indicator of cell proliferation, was highly expressed in the ISX9@TBNPs and ISX9 groups compared to their respective control groups (Fig. 5a).

The western blot analysis revealed that treatment with ISX9@TBNPs led to increased levels of various Wnt/β-catenin pathway and hair-related factors, including active β-catenin, β-catenin, LEF1, Axin2, survivin, SOX2, LGR5, K17 and Ki67, compared to topical ISX9 (Fig. 5b and c). These factors play important roles in regulating the Wnt/β-catenin pathway, which is known to be critical for hair regrowth. Furthermore, gene expression analysis showed significantly higher expression levels of several Wnt-targeted (Axin2, LEF1, survivin, Fibronectin), Stemness marker (LGR5, SOX2, Twist1, OCT4) and hair-related genes (VEGF, HGF and IGF-1) in mice treated with ISX9@TBNPs compared to other treatment groups (Fig. 5d). These findings suggest that ISX9@TBNPs is an effective method for inducing the expression of epidermal proteins, HF stem cell markers, and the Wnt/β-catenin pathway for hair regeneration.

To assess the potential toxicity of TBNPs and ISX9@TBNPs, histological examination of the major organs (liver, kidney, spleen, heart, lung) was conducted in mice after treatment (Fig. S9). The results showed no apparent toxicity in any of the examined organs, indicating that these nanoparticles are safe for use *in vivo*. Overall, the findings suggest that the improved hair density and regrowth observed in mice treated with ISX9@TBNPs are likely due to the combination of temperature sensitivity and sustained release of ISX9, which leads to enhanced expression of hair-related factors and activation of the Wnt/β-catenin pathway.

## 4 Conclusion

The present study demonstrated successful delivery of a hydrophobic drug to targeted tissues using modified biopolymer-based thermos-responsive nanoparticles (NPs) (Fig. 1). The chitosan (CS) was modified with glycidyl methacrylate (GMA) to introduce alkene groups on the polysaccharide, which was then copolymerized with N-isopropylethyl methacrylate (NIPAAm) to obtain the poly(NIPAM-*co*-GMA@CS) shell polymer. The self-assembly of the PF127 block copolymer facilitated the solubility of hydrophobic ISX9 in the core, and the thermos-responsive shell polymer allowed sustained release of the drug. The drug-loaded TBNPs with a hydrodynamic diameter of 100–250 nm demonstrated efficient and controlled drug release at 32 ± 2 °C compared to 25 ± 2 °C.

Compared to current widely used androgenetic alopecia treatments, the dual-functionalized ISX9@TBNPs system has the potential to induce hair regeneration with enhanced efficacy, precision, and convenience.

## Credit author statement

D L, M S and S S designed the research; S S, M S, J S, Q S, H D, V W X, S L, B D performed experiments; S S, G Z, M S, G Z and D L carried out the data analysis. D L, M S, S S, G Z, F J S made critical comments on the manuscript. S S, M S, G Z and D L wrote and edited the manuscript.Sapna Sayed & Mehdihassan Shekh: Conceptualization, Methodology, Software, Validation, Formal analysis, Investigation, Resources, Data curation, Writing – original draft, Writing – review & editing, Visualization, Supervision, Project administration, Funding acquisition, Jiaxing Song & Qi Sun: Formal analysis, Investigation Resources, Data curation, Software, Validation, Formal analysis, Han Dai, Vivian Weiwen Xu & Shanshan Liu: Formal analysis, Investigation, Resources, Bing Du, Guangqian Zhou, & Florian J. Stadler: Data curation, Writing – original draft, Writing – review & editing, Visualization, Guangming Zhu: Methodology, Software, Validation, Formal analysis, Investigation, Resources, Data curation, Writing – original draft, Writing – review & editing, Visualization, Supervision, Project administration, Funding acquisition, Desheng Lu: Conceptualization, Methodology, Software, Validation, Formal analysis, Investigation, Resources, Data curation, Writing – original draft, Writing – review & editing, Visualization, Supervision, Project administration, Funding acquisition

## Declaration of competing interest

The authors declare that they have no known competing financial interests or personal relationships that could have appeared to influence the work reported in this paper.

## Data Availability

Data will be made available on request.
