# Supplementary material for: ISX9 loaded thermoresponsive nanoparticles for hair follicle regrowth
Source: Mater Today Bio. 2023 Nov 3;23:100849. doi: 10.1016/j.mtbio.2023.100849 (PMC10682119; doi:10.1016/j.mtbio.2023.100849)
Supplement: Multimedia component 1 [file mmc1.pdf]

Supplementary material

Supplemental Table S1 Primers used in this study(Mouse)

**Primer ID**	**Sense (5’-3’)**	**Anti-sense (5’-3’)**
AXIN2	AACCTATGCCCGTTTCCTCTA	GAGTGTAAAGACTTGGTCCACC
LEF1	TTGATGTCGGCTAAGTCGCC	GCCACCGATGAGATGATCCC
Fibronectin	TTTGCTGCGATTGGTGACATT	ATGCCAAATCTTGCGGAGAAT
Survivin	GAGGCTGGCTTCATCCACTG	CTTTTTGCTTGTTGTTGGTCTCC
GAPDH	TGGATTTGGACGCATTGGTC	TTTGCACTGGTACGTGTTGAT
LGR5	ATGTGGTTGGCATCTAGGCG	ACATTCCCAAGGGAGCGTTC
Sox2	GCGGAGTGGAAACTTTTGTCC	GGGAAGCGTGTACTTATCCTTCT
OCT4	CGAAGCGACAGATGGTGGTC	AGAGGATCACCTTGGGGTACA
Twist1	GGACAAGCTGAGCAAGATTCA	CGGAGAAGGCGTAGCTGAG
HGF	GTCCTGAAGGCTCAGACTTGGT	CCAGCCGTAAATACTGCAAGTGG
VEGF	TGCAGATTATGCGGATCAAACC	TGCATTCACATTTGTTGTGCTGTAG
IGF	CTGGTCCTGTGTCCCTTTGC	GGACGGGGACTTCTGATC
