# Supplementary material for: ISX9 loaded thermoresponsive nanoparticles for hair follicle regrowth
Source: Mater Today Bio. 2023 Nov 3;23:100849. doi: 10.1016/j.mtbio.2023.100849 (PMC10682119; doi:10.1016/j.mtbio.2023.100849)
Supplement: Multimedia component 2 [file mmc2.docx]

ISX9 loaded Thermoresponsive Nanoparticles for Hair Follicle Regrowth

Sapna Sayed^1#^, Mehdihassan Shekh^2#^, Jiaxing Song^1, 3^, Qi Sun^1^, Han Dai^1^, Vivian Weiwen Xue^1^, Shanshan Liu^1^, Bing Du^2^, Guangqian Zhou^1^, Florian J. Stadler^2^, Guangming Zhu^2*^, Desheng Lu^1^**

^1^ Guangdong Provincial Key Laboratory of Regional Immunity and Diseases, International Cancer Center, Department of Pharmacology, Shenzhen University Medical School, Shenzhen, China

^2^ New Energy Materials Laboratory, College of Materials Science and Engineering, Shenzhen University, Shenzhen, China

^3^Medical Scientific Research Center, Life Sciences Institute, Guangxi Medical University, Nanning, China.

* To whom correspondence may be addressed. Desheng Lu and Guangming Zhu, Shenzhen University Medical School, Shenzhen 518060, Guangdong, China.

#Sapna Sayed (M.phil., Ph.D.,) and Mehdihasan I. Shekh (M. Sc., Ph.D.,), both are sharing the equal contribution in this research work.

Email: [gzhu@szu.edu.cn](mailto:gzhu@szu.edu.cn) & [delu@szu.edu.cn](mailto:delu@szu.edu.cn)

**Supporting Data**

**(a)**

**Figure S1.** (a) Synthesis route of modification of CS with GMA followed by polymerization with N, N-isopropyl acrylamide (NIPAAm)


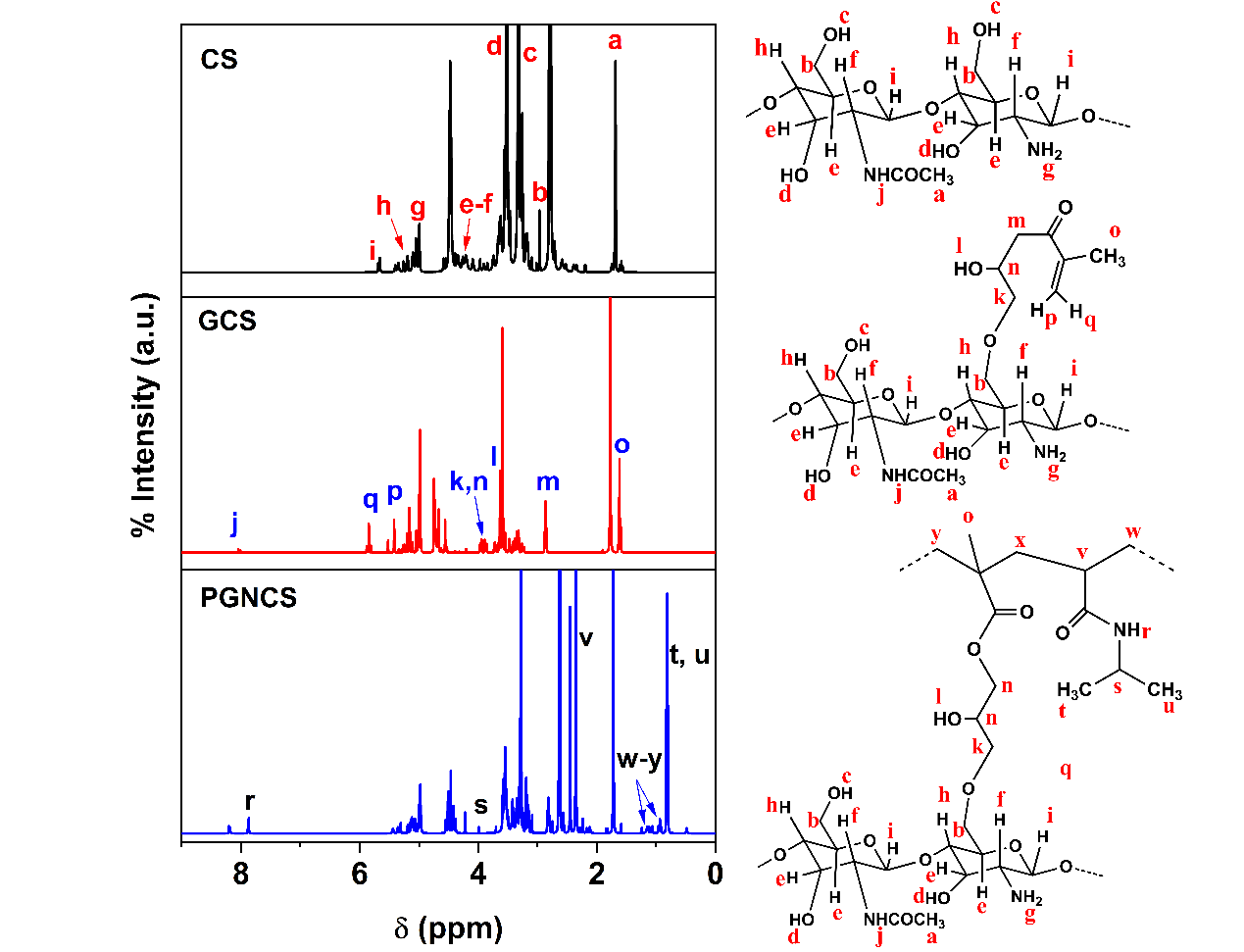


**Figure S2.** (a) ^1^H-NMR of Chitosan (CS); GCS; PGNCS (collected in 0.4 M CH3COOH/D_2_O)

**
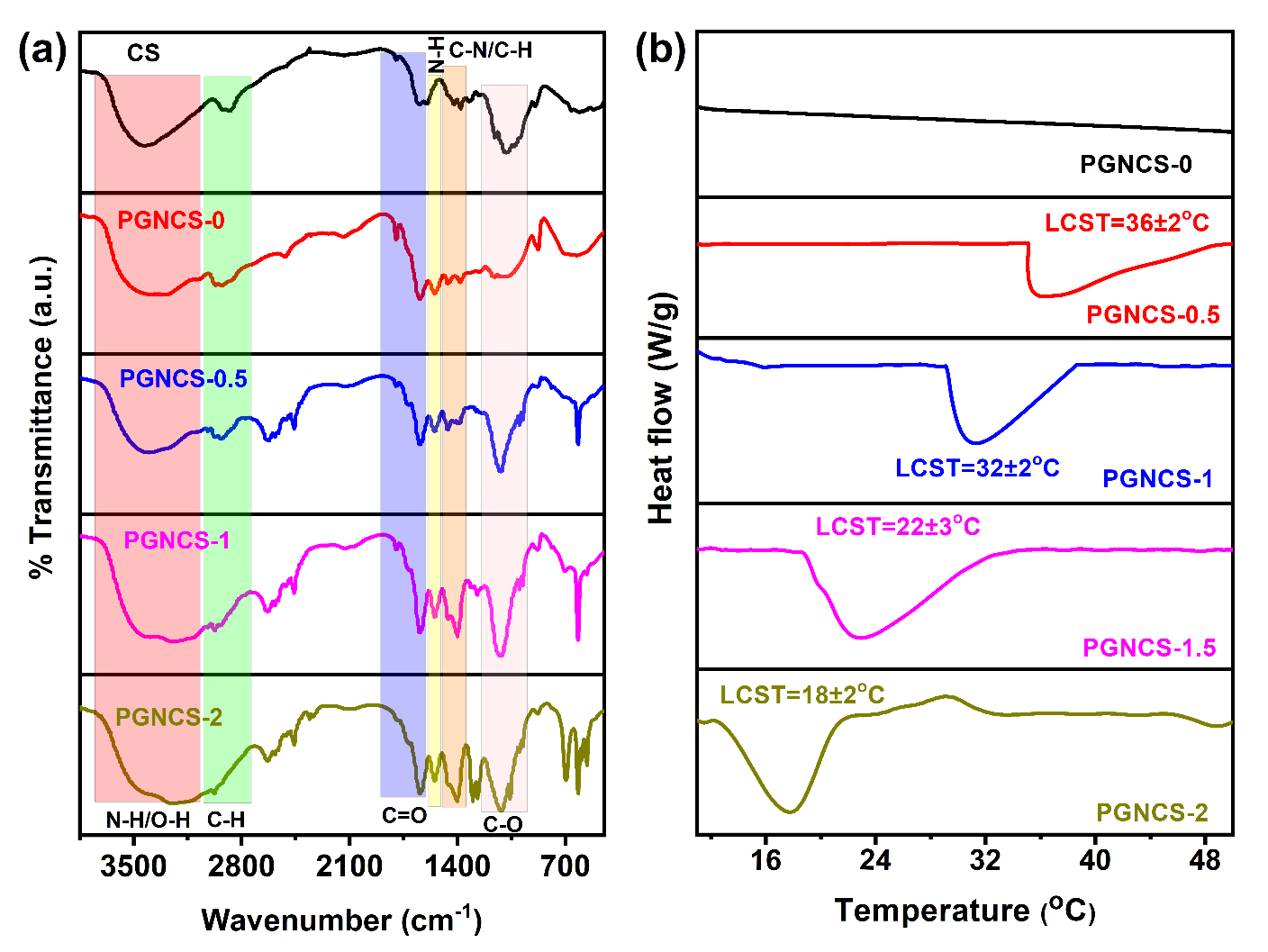
**

**Figure S3.** (a) FTIR spectra of raw materials, and (b). DSC thermograms of raw materials


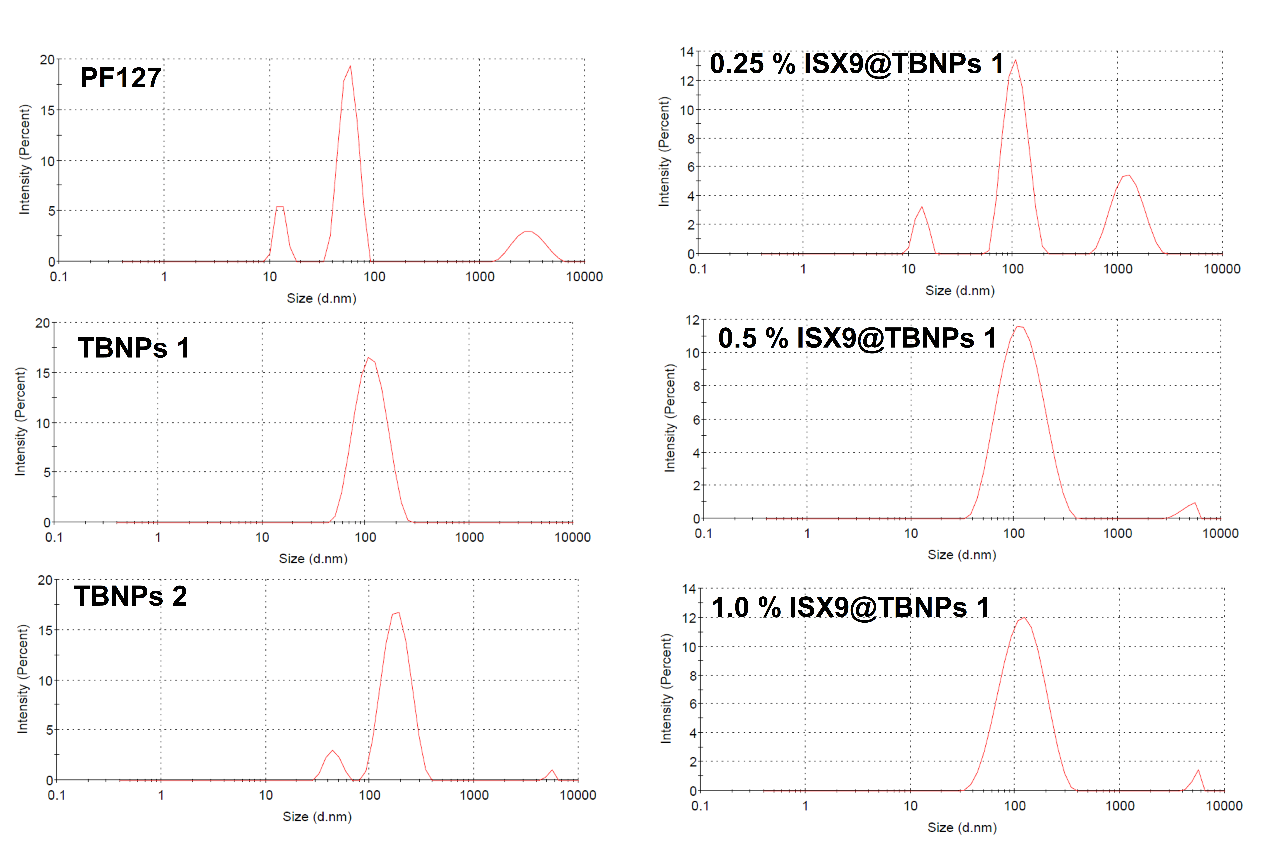


**Fig. S4** Size distribution plots of different nanoparticles


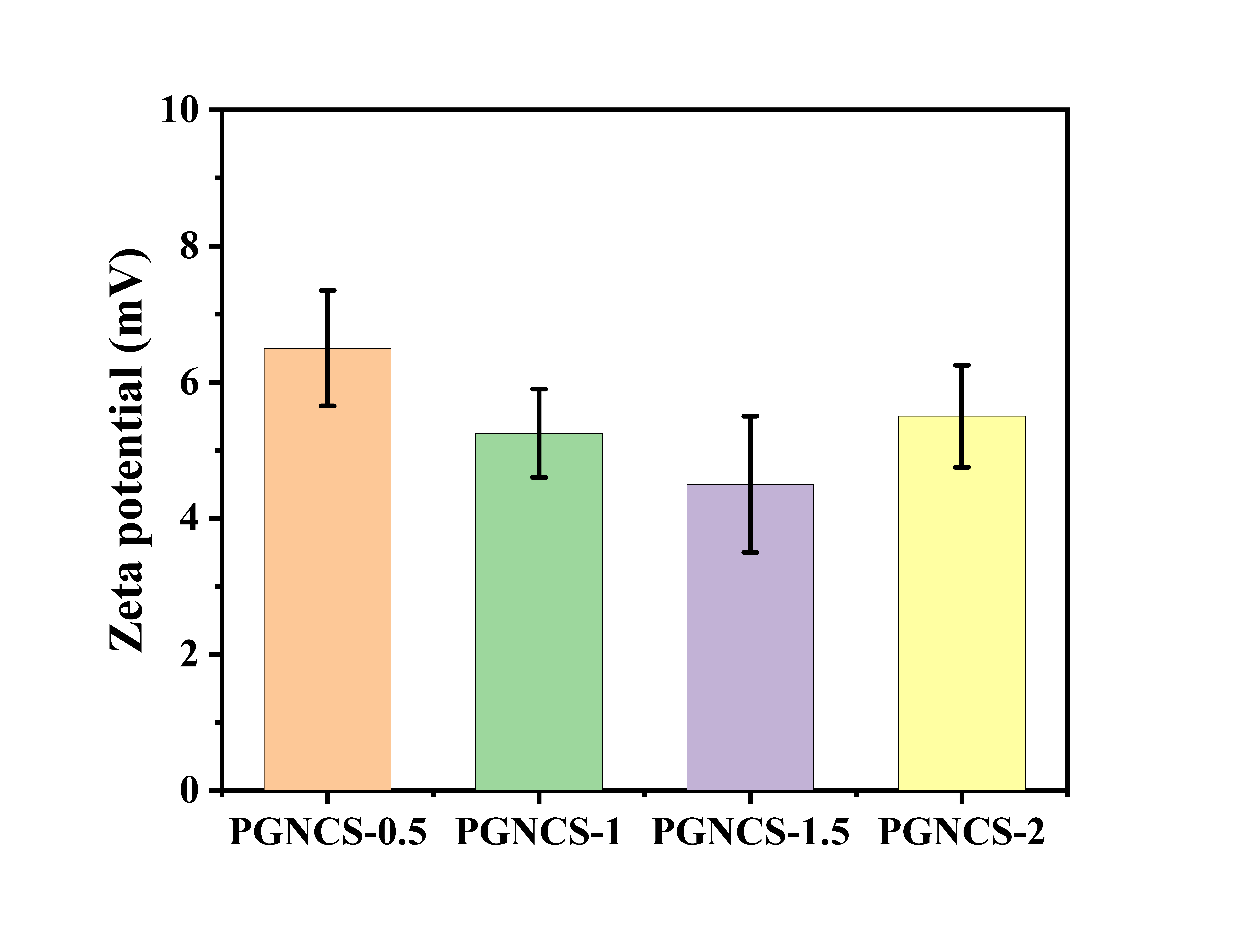


**Figure S5.** (a) Zeta potential of different poly(NIPAAm(x)-co.-GMA)@CS (where X= 1:0.5; 1:1; 1:1.5 & 1:2)


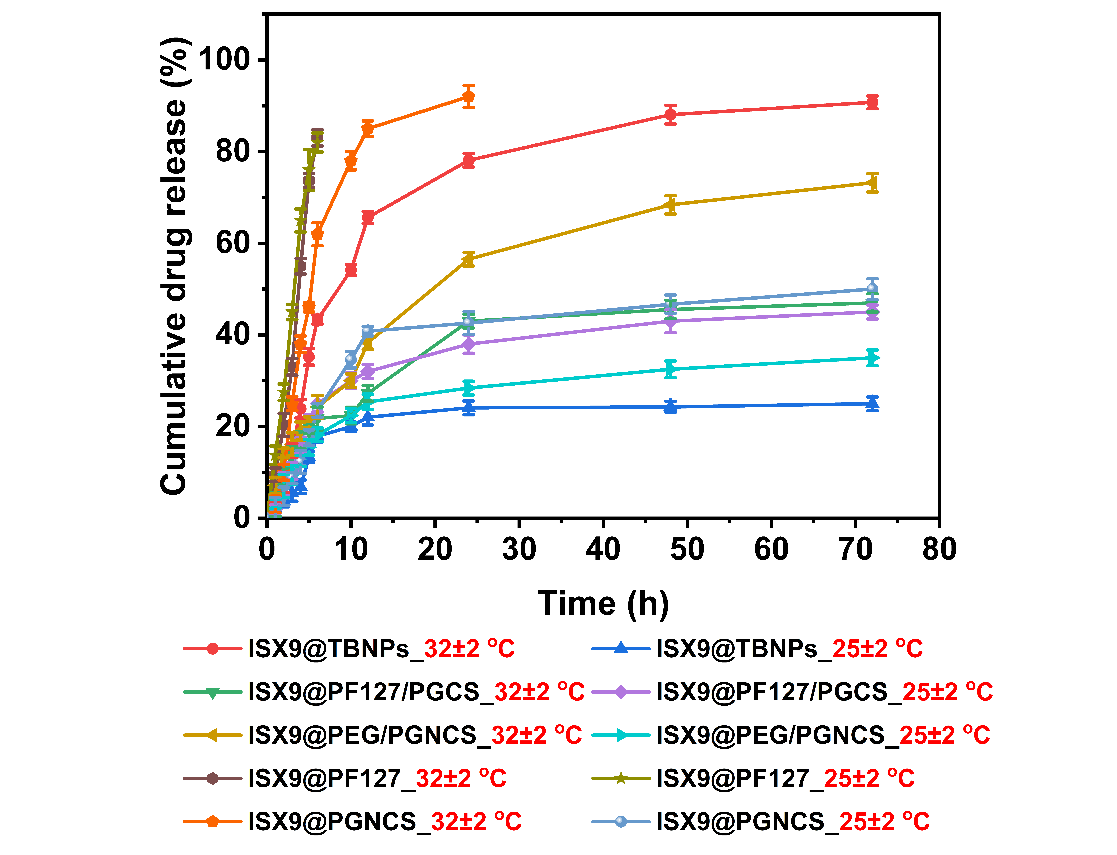


**Figure S6**. (a) Cumulative drug release profiles of different drug carriers. (**Note:** ISX9@PF127/PGNCS is 1 wt % of ISX9 loaded TBNPs-1 nanparticles while ISX9@PF129/PGCS is 1 wt % of ISX9 loaded poly(glycidyl methacrylate@Chitosan) nanoparticles.

**(a)**


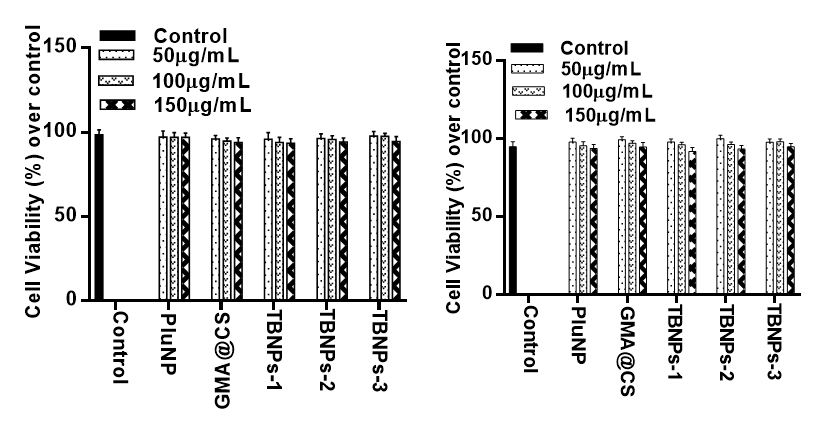


**(b)**

**Figure S7.** In vitro cytotoxicity of the nanoparticles in (a). HaCaT keratinocyte cells, and (b)NIH3T3 cells cytotoxicity was measured using a cell counting kit-8 (CCK-8) assay. Data represents means and standard errors of at least a triplicate determination.

**
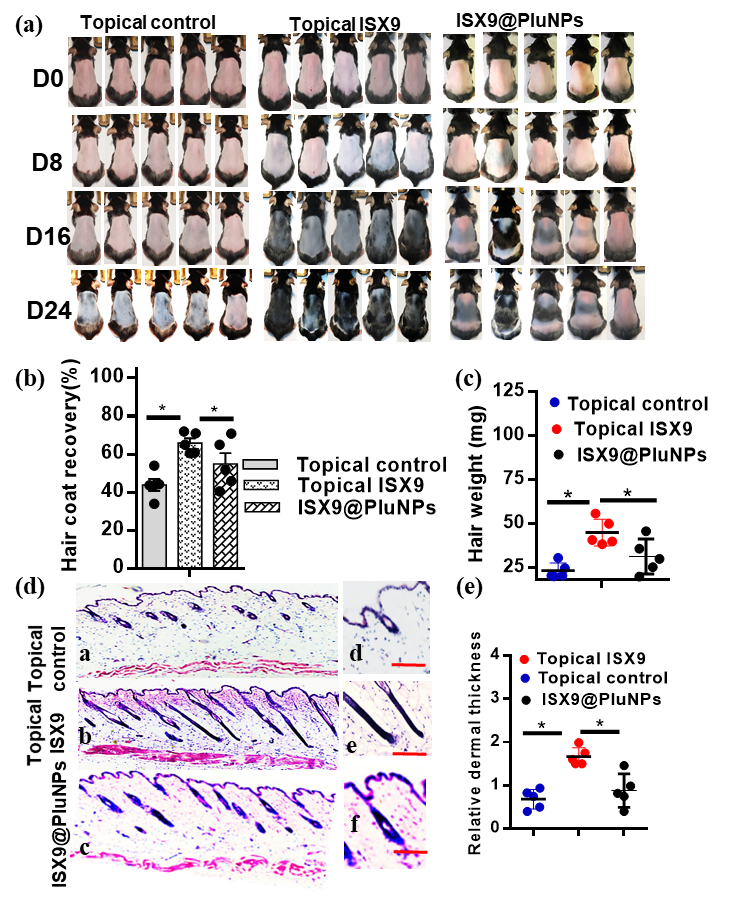
**

**Figure S8. *In vivo* effectiveness of ISX9 loaded nanoparticles in C57BL/6 mice.** C57BL/6J mice in the telogen phase (7 weeks old, female) were depilated. Topical control, topical ISX9, or ISX9@PLuNPs, were topically applied thrice a week to the dorsal skin for 24 days (n = 5 per group). (a) Representative photos of mice showed hair regrowth on 0, 8, 16, 24th day treated with different combinations: topical control, topical ISX9, or ISX9@PluNPs. (b) Quantitative measurements of hair coat recovery at the designated area at 24th day. (c) Gross analyses of weight of regrown hair in different groups treated at 24th day. Data shown were representative of five independent experiments (*n* = 5). Values are means ± *SD*. **P* < 0.05, significantly different from the topical control. (d) H&E stained dorsal skin at 24th day in different groups (a, b, c). Also d, e, and f were the enlargements of the framed area in a, b, and c, respectively. Scale bar = 100 μm. (e) Relative dermal thickness among mentioned groups at 24th day. Data are expressed as mean ± *SD* (n = 5). *P < 0.05 significantly different to control.

**(a)**

**Heart**

**Lungs**

**Liver**

**Spleen**

**Kidney**


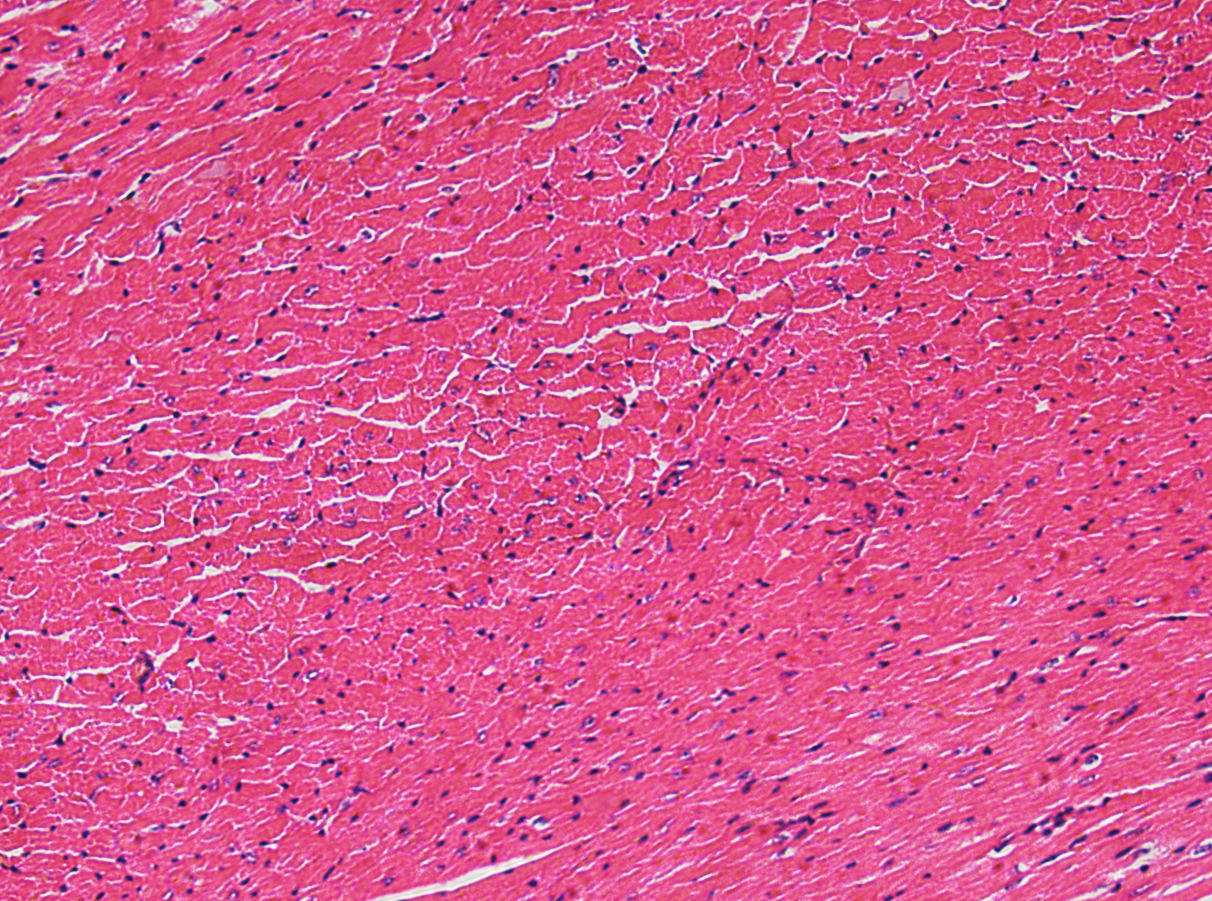

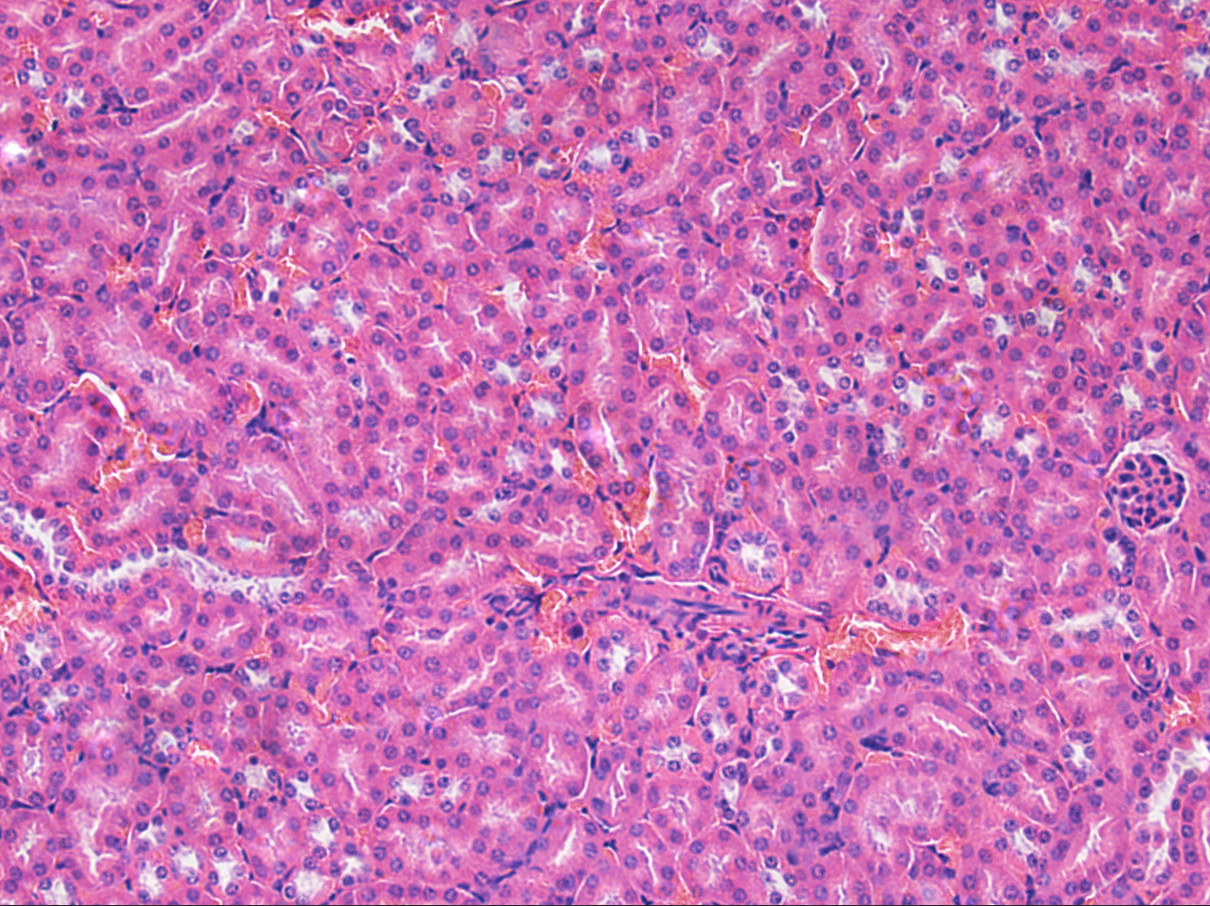

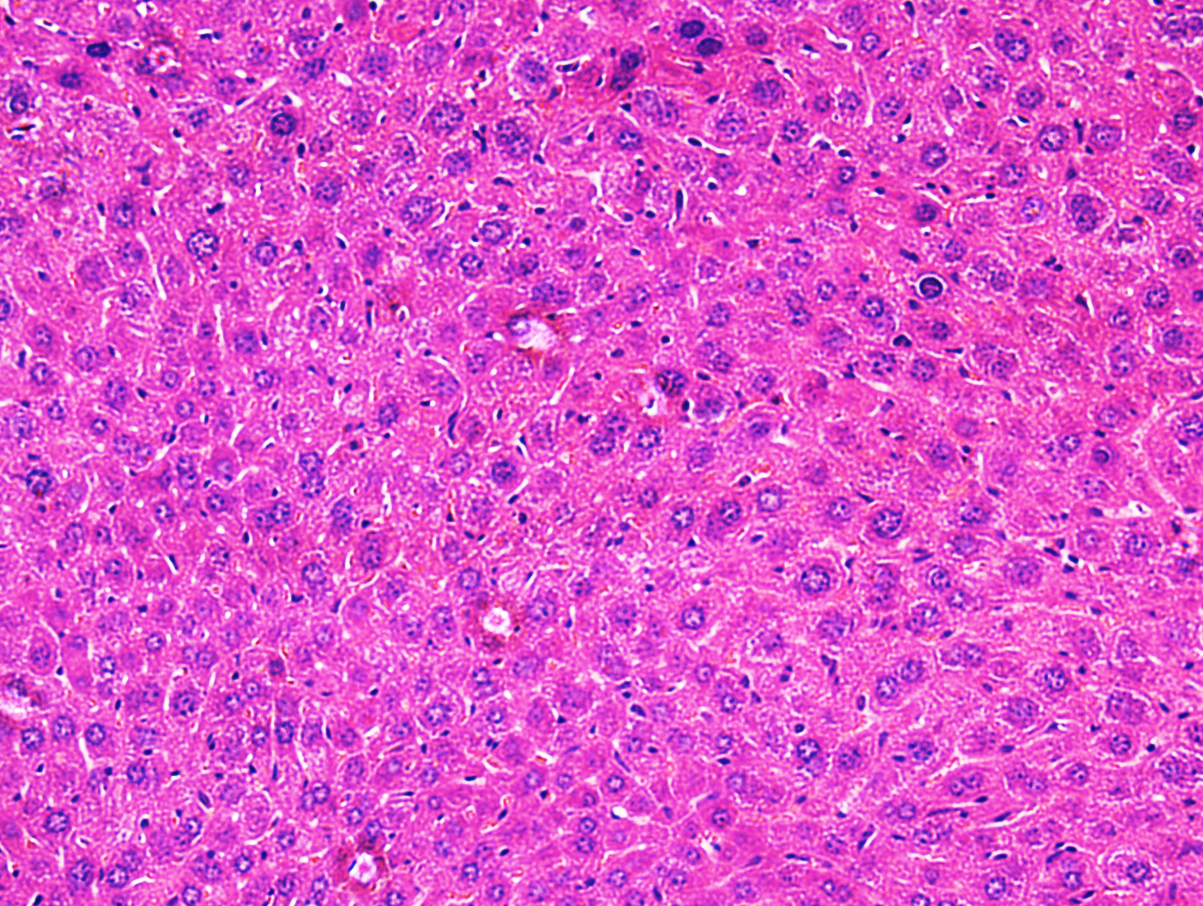

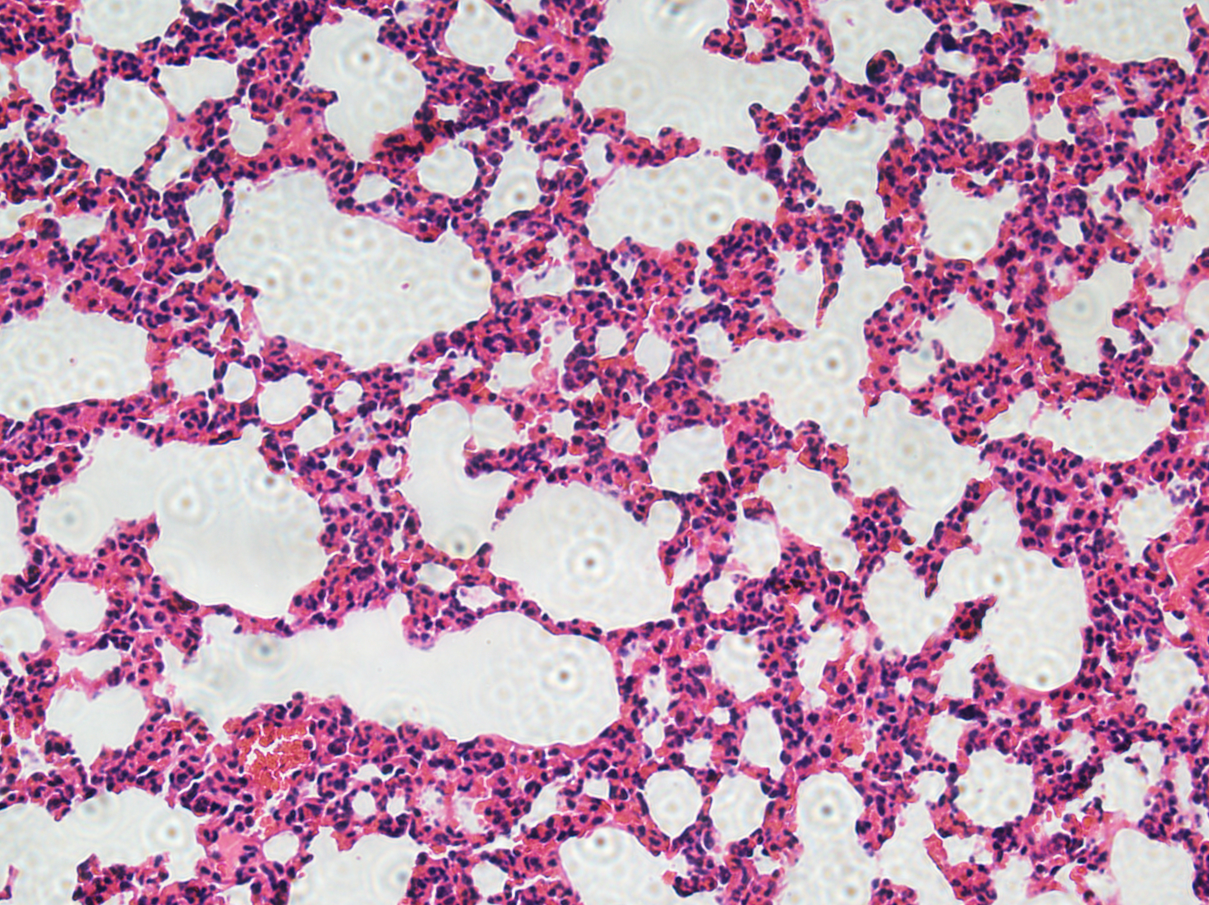

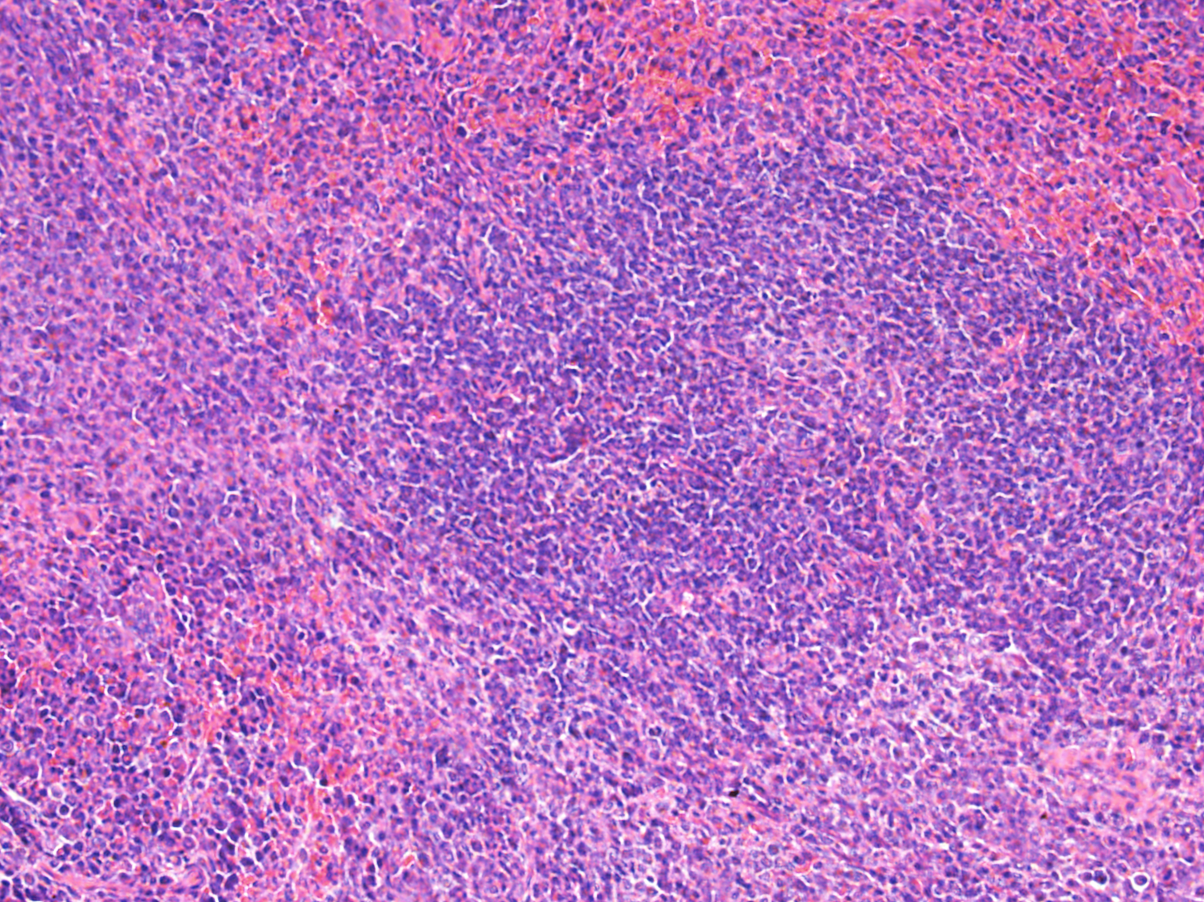

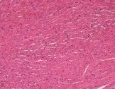

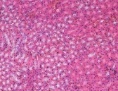

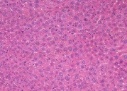

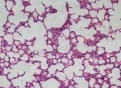

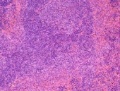

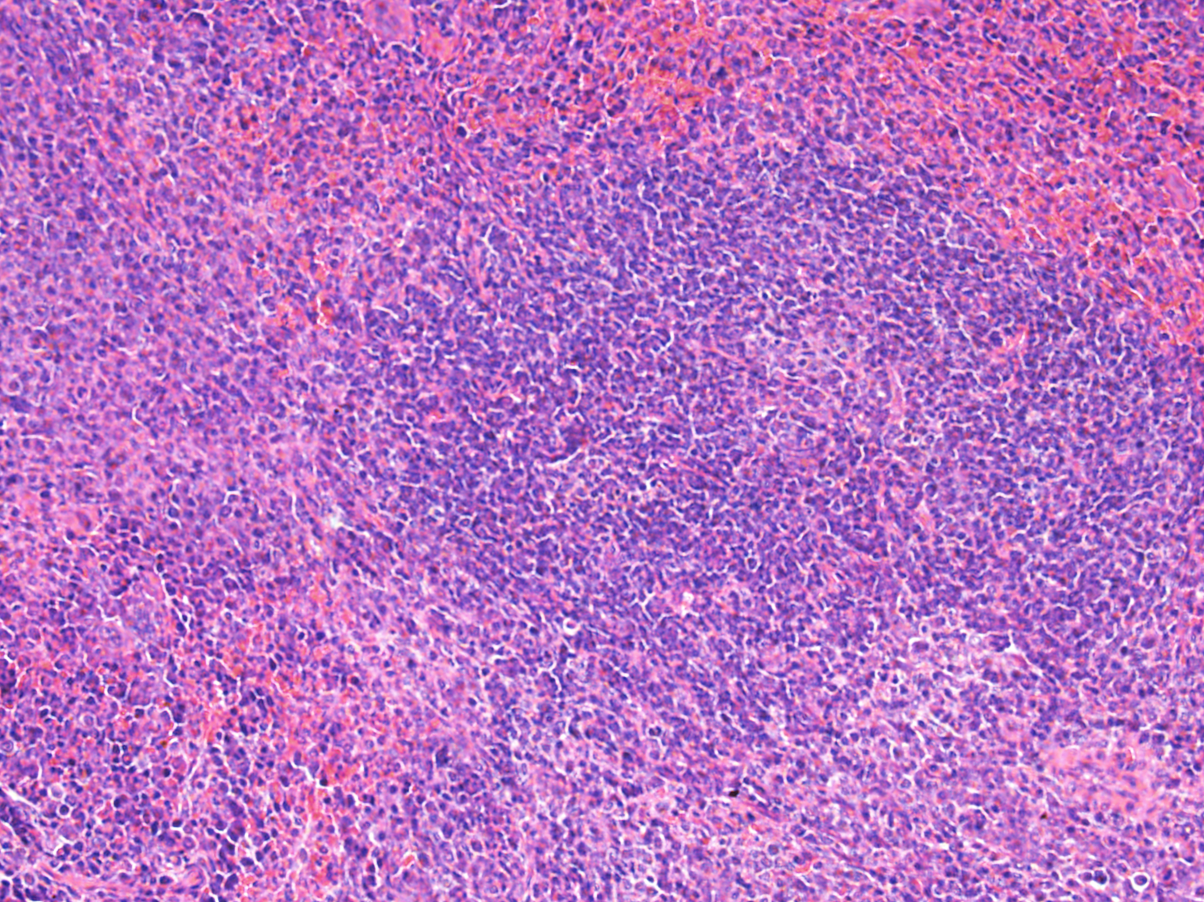


**TBNPs**

**ISX9@TBNPs**

**Figure S9. *In vivo* systemic toxicity evaluation.** C57BL/6J mice in the telogen phase were depilated, TBNPs or ISX9@TBNPs, were topically applied thrice a week to the dorsal skin for 24 days (n = 5 per group). (a). H&E staining of major organs (including heart, liver, spleen, lung and kidney) after topically applied TBNPs or ISX9@TBNPs thrice a week to the dorsal skin for 24 days after depilation. Scale bar: 100 μm.
